# Mitochondria in COVID-19: from cellular and molecular perspective

**DOI:** 10.3389/fphys.2024.1406635

**Published:** 2024-06-21

**Authors:** Michał Rurek

**Affiliations:** Department of Molecular and Cellular Biology, Institute of Molecular Biology and Biotechnology, Faculty of Biology, Adam Mickiewicz University, Poznań, Poland

**Keywords:** cellular signaling, COVID-19, mitochondrial biogenesis, mitogenome, protein-protein interactions, respiration, SARS-CoV-2

## Abstract

The rapid development of the COVID-19 pandemic resulted in a closer analysis of cell functioning during β-coronavirus infection. This review will describe evidence for COVID-19 as a syndrome with a strong, albeit still underestimated, mitochondrial component. Due to the sensitivity of host mitochondria to coronavirus infection, SARS-CoV-2 affects mitochondrial signaling, modulates the immune response, modifies cellular energy metabolism, induces apoptosis and ageing, worsening COVID-19 symptoms which can sometimes be fatal. Various aberrations across human systems and tissues and their relationships with mitochondria were reported. In this review, particular attention is given to characterization of multiple alterations in gene expression pattern and mitochondrial metabolism in COVID-19; the complexity of interactions between SARS-CoV-2 and mitochondrial proteins is presented. The participation of mitogenome fragments in cell signaling and the occurrence of SARS-CoV-2 subgenomic RNA within membranous compartments, including mitochondria is widely discussed. As SARS-CoV-2 severely affects the quality system of mitochondria, the cellular background for aberrations in mitochondrial dynamics in COVID-19 is additionally characterized. Finally, perspectives on the mitigation of COVID-19 symptoms by affecting mitochondrial biogenesis by numerous compounds and therapeutic treatments are briefly outlined.

## Graphical Abstract

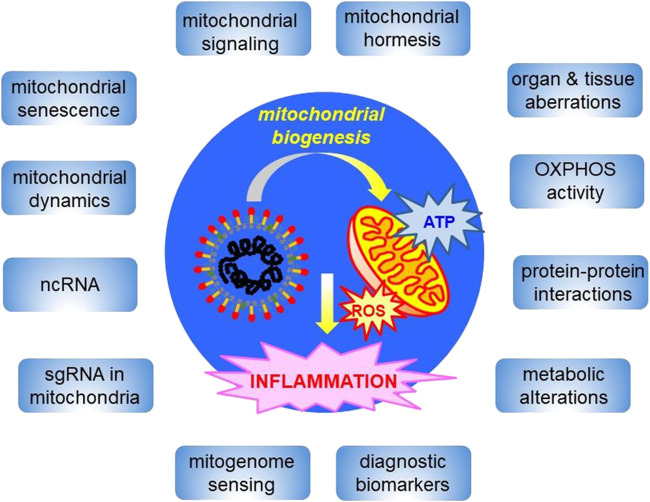

## 1 Introduction

SARS-CoV-2 (severe acute respiratory syndrome-coronavirus-2), an RNA-containing virus belonging to the Coronaviridae family, is a causative agent of COVID-19 (from CoronaVirus Disease). COVID-19 has spread throughout the world and resulted in 775,431,269 total cumulative cases and 7,047,741 total cumulative deaths reported to the World Health Organization (World Health Organization, 2024), which are not equally distributed across various states. Based on those data, the United States, Brazil, India, Russia, Mexico, United Kingdom, Peru, Italy, Germany, and France had the highest mortality due to SARS-CoV-2 infection. According to World Health Organization data, in the US, 103,436,829 and 1,186,984 cumulative numbers of COVID-19 detected cases and deaths (5 May 2024) have been noted, respectively. In contrast, in some small and/or island areas (due to their minor population), particularly on the Pitcairn, Holy See and Tokelau islands, the total cumulative cases were 4, 26 and 80, respectively. The COVID-19 outbreak has rapidly accelerated to a global pandemic, with more than 83,832,334 SARS-CoV-2 infections and 1,824,590 deaths worldwide until the end of 2020 (The American Journal of Managed Care, 2021).

The COVID-19 pandemic has challenged the life of the world population. It started in Wuhan (China), where a growing number of pneumonia cases appeared in December 2019. The main symptoms were initially high fever, persistent cough, fatigue, headache, and muscle aches, indicating bioenergetic deficiencies; furthermore, COVID-19 worsened patient mental health and sleep quality (Huang and Zhao, 2020). From the beginning of the pandemic, various models for dynamic COVID-19 transmission were discussed, however, no evidence of animal-to-human transmission of the novel virus was proposed, leading to the hypothesis that the virus spread among humans occurred through an intermediate host (Shahid et al., 2020).

Due to the complexity of mitochondrial organization and functions, a significant influence of COVID-19 on mitochondrial biogenesis should be expected. In fact, mitochondria belong to the compartments most severely affected by SARS-CoV-2 infection. Based on the current research, there is a need for an updated review where such dependencies will be exhaustively summarized. Therefore, the objective of this review is to characterize complex relationships between SARS-CoV-2 and human mitochondria at tissue, cellular, and molecular levels. Multiple impairments in mitochondrial biogenesis are discussed within system- and tissue-specific contexts. In this review, severe alterations in mitochondrial metabolism and gene expression pattern are focused. Numerous activities and interactions of SARS-CoV-2 proteins with host mitochondrial proteins are also summarized. The presence of SARS-CoV-2 subgenomic RNA (sgRNA) within mitochondria, mitogenome alterations and mitochondrial motility and dynamics in COVID-19 are exhaustively discussed. Finally, current therapeutic approaches related with the usage of various compounds alleviating COVID-19 symptoms and maintaining mitochondrial hormesis are outlined.

## 2 SARS-CoV-2, its genome, key proteins, and replication

Coronaviridae family consists of two subfamilies (Orthocoronavirinae and Letovirinae), with 46 virus species in total. Furthermore, Letovirinae contains only α-letovirus, and four types of coronaviruses (α-, β- γ- and δ-) belong to the Orthocoronavirinae subfamily (Leao et al., 2022; Mavrodiev et al., 2022). While γ- and δ-coronaviruses infect mainly birds, but some of them also mammals, α- and β-coronaviruses infect only mammals (Wang et al., 2022). Until now, few coronaviruses that infect human cells (HCoV) have been identified, including HCoV-HKU1, HCoV-OC43, HCoV-229E, HCoV-NL63, SARS-CoV-1, SARS-CoV-2, and Middle East Respiratory Syndrome coronavirus (MERS-CoV). They cause severe respiratory diseases that can be potentially lethal or lead to a decreased quality of life in post-acute COVID-19 syndrome. At the early onset of the pandemic, various SARS-CoV-2 subtypes were characterized by both high contagiousness and virulence (Malik et al., 2022).

**FIGURE 1:**
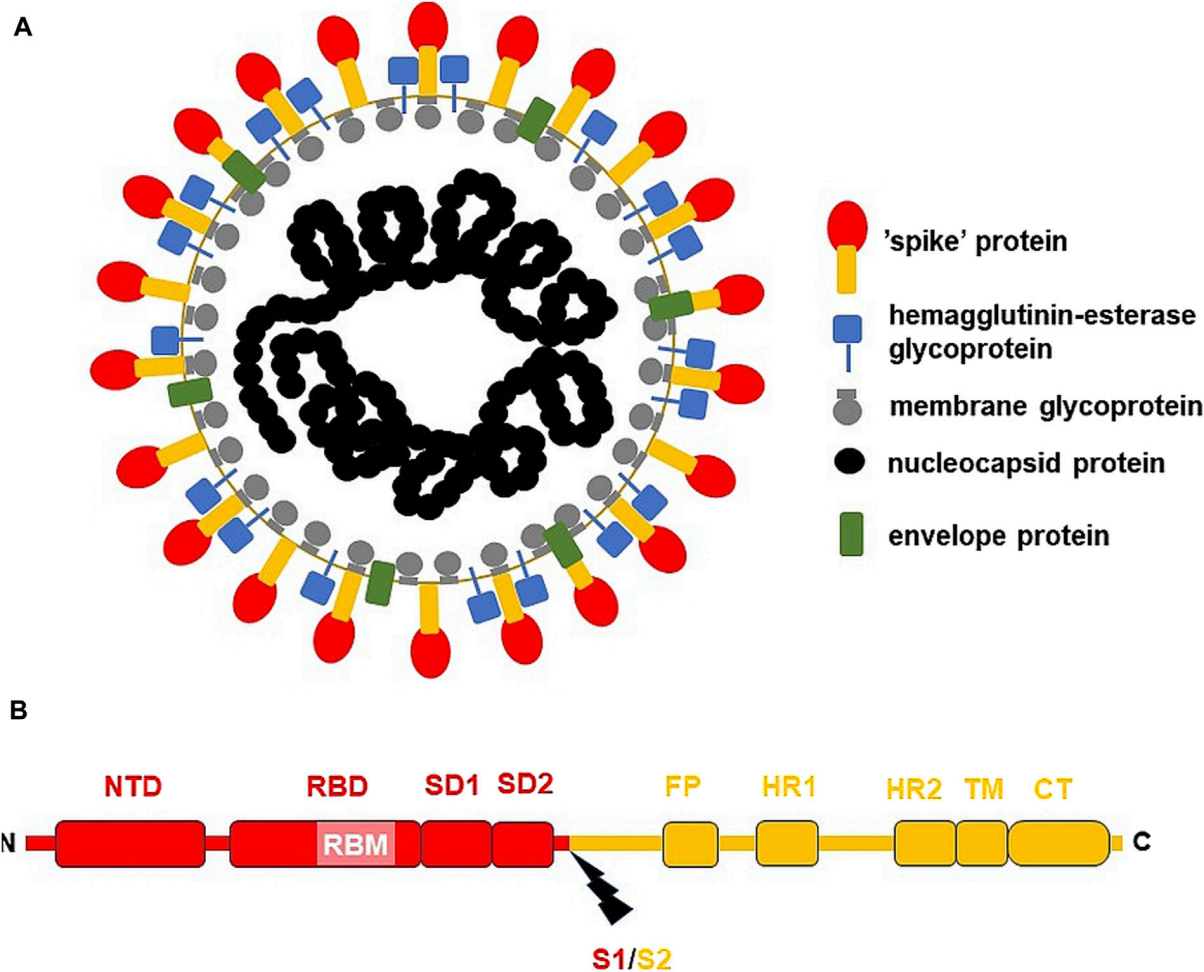
The general structure of SARS-CoV-2 virion and S-protein. **(A)** SARS-CoV-2 virion; **(B)** S-protein from USA/WA1 SARS-CoV-2 strain. The subunit S1 was depicted red, while subunit S2 (the membrane anchoring subunit) in orange. CT, cytoplasmic tail; FP, fusion peptide, HR1, heptad repeat 1; HR2, heptad repeat 2; NTD, N-terminal domain; RBD, receptor binding domain; RBM, receptor binding motif within RBD; S1/S2, the cleavage site (*indicated by thunderbolt mark*); SD1, subunit domain 1; SD2, subunit domain 2; TM, transmembrane domain.

SARS-CoV-2 belongs to large β-coronaviruses, which contain loosely folded single-stranded positive-polarity RNA as genomic RNA (gRNA), and the protein envelope (Figure 1). Coronavirus virions range from 118 to 140 nm in diameter, and their genomes from 25 to 32 kbs in size (Payne, 2017). The SARS-CoV-2 genome forms typical secondary and tertiary structures, containing both long arms and loops. Notably various methods, including NaI/DMS crosslinking, allowed for advanced analyses of these structures (Yang et al., 2020).

**FIGURE 2:**
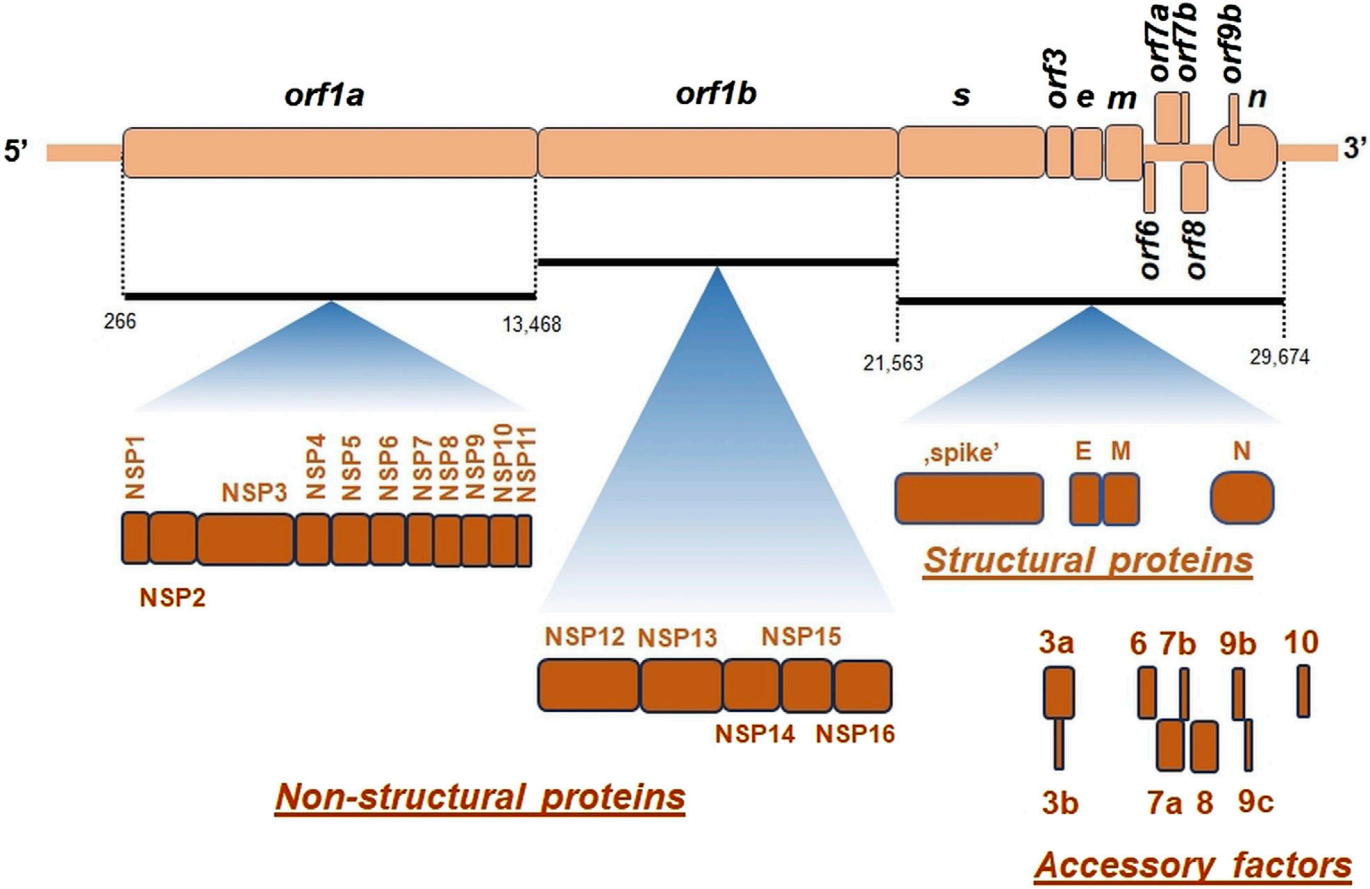
The general organization of SARS-CoV-2 genome. *orf*, open reading frame; *e*, gene encoding envelope protein; *m*, gene for membrane protein; *n*, gene encoding nucleocapsid protein; NSP, non-structural proteins; *s*, gene encoding “spike” protein. On genomic scheme, positions of coding sequences for small accessory proteins as well as for structural and non-structural proteins (*below*) were indicated. Key nucleotide coordinates (in nt) separating various *orfs* were also shown.

SARS-CoV-2 virion contains an envelope composed of numerous proteins, some of which interact with the membrane of target cells (Woo et al., 2023). These proteins include “spike”- (S-), membrane- (M-) and envelope- (E−) proteins, as well as hemagglutinin esterase (HF) dimers, and the internal nucleocapsid- (N-) protein interacting with gRNA. SARS-CoV-2 genome contains multiple open reading frames (*orfs*) coding for both nonstructural (NSP1-NSP16), structural (e.g., E−, M-, S-, N-), and other proteins (Figures 1, 2). Among those *orfs*, the most prominent are *orf1a* and fused *orf1a/1b* encoding, respectively, pp1a and pp1b polyproteins for multiple non-structural proteins that form the replication-transcription complex. Products of expression of other *orfs*, for example, *orf3a*, *orf6*, *orf7a*, *orf7b*, *orf8a*, *orf9b*, *orf9c* and *orf10* interact with a plethora of mitochondrial proteins (Figure 2). The main activities and interactions of all identified NSPs are shown in Table 1 and addressed in detail in Chapter 6.

**TABLE 1:** Most important activities of SARS-CoV-2 non-structural proteins.

NSP	Functions
NSP1	interacts with host ribosomes, thereby inhibiting host protein synthesis; host IFN signaling suppressor
NSP2	interacts with ribosomes and replication-transcription complexes to enable viral transcription and translation; suppresses translation of host transcripts; prohibitin inhibitor; GIGYF2, VDAC, Hsp70, STOML and vATPase interactors; with NSP4 affects mitochondrial Ca^2+^ homeostasis (IP3R)
NSP3	encoded by papain-like protease domain; undergoes autohydrolysis to form NSP1-3 proteins; NSP3 contains several other domains with possibly distinct functions: a ubiquitin-like domain, an X domain, a nucleic acid-binding domain, a domain unique to the SARS virus, a transmembrane domain, and a Y1-3 domain; essential in RNA metabolism (component of replication-transcription complexes) and blocking host innate immune response; causes ER membrane bending with NSP4 and NSP6; essential for SARS-CoV-2 virulence
NSP4	causes ER membrane bending with NSP3 and NSP6; with NSP2 affects mitochondrial Ca^2+^ homeostasis (IP3R) and TIM activity; causes ER membrane bending with NSP3 and NSP6; regulates the level of the negative regulator of cell proliferation
NSP5	hydrolyzes pp1a and pp1ab polyproteins at 11 amino acid positions to release mature NSP4–NSP16 proteins; targeting RIG-I and MAVS signalosome complex and MAGED2 cleavage; block host innate immune response
NSP6	causes ER membrane bending with NSP3 and NSP4; zippers ER membranes and establishes the connectors; participates in DMV biogenesis
NSP7	interacts with NSP12 to increase RNA polymerase processivity; interacts with accessory proteins for OXPHOS biogenesis; NSP8 also interacts with MRPs and leads to the autophagosome accumulation and the incomplete mitophagy
NSP8	
NSP9	inhibits NSP12 nucleotidyltransferase activity
NSP10	cofactor to NSP14 and NSP16 proteins
NSP12	virus RNA-dependent RNA polymerase and nucleotidyltransferase
NSP13	helicase activity
NSP14	exoribonuclease and N7-guanylmethyltransferase activity
NSP15	uridine-specific endoribonuclease
NSP16	2′-O-methyltransferase, mediating cap biogenesis at the 5′end of mRNA

There are at least few additional open reading frames between the structural genes of the β-coronaviruses, encoding additional proteins (Hu et al., 2021). SARS-CoV-2 genome does not contain *orf3b* gene, which differentiates it from SARS-CoV-1 (Singh et al., 2020a). Interestingly, *orf10* is also uniquely expressed in SARS-CoV-2 and encodes a protein that can suppress the interferon (IFN) response (Figure 2) (Li et al., 2022).

Once the virus gRNA enters the cell, *orf1a* and *orf1b* genes are immediately expressed and a replication-transcription complex is formed. Viral gRNA is released from endosomes to the cytoplasm, where the replication cycle starts (Shanmugaraj et al., 2020). gRNA synthesis occurs within DMVs and the vesicle-tubular network of coiled membranes (CMs), known as “virus replication organelles.” Replication-transcription complexes begin to produce gRNA progeny molecules and mRNA that encode several structural proteins. In general, the expression of the SARS-CoV-2 proteins in infected cells depends mainly on the translation of gRNA and sgRNA. Coronaviruses (including SARS-CoV-2) use non-canonical translation mechanisms to enhance their coding capacity and adjust the expression levels of individual viral proteins. For example, translation of pp1ab depends on the presence of the programmed ribosomal frame shift (PRF) at position −1 upstream of *orf1a* termination codon, thus extending the pp1a open reading frame pp1a from *orf1b*. As a result, up to 2-times overproduction of the protein encoded by *orf1a* relative to *orf1b* was detected (Malone et al., 2022). Thus, viral RNA enables rapid translation of selected reading frames, while newly synthesized NSPs regulate replication and transcription of the SARS-CoV-2 genome. All translated proteins and synthesized genomic copies of the coronavirus are transported to the endoplasmic reticulum (ER) and the Golgi apparatus (GA), and new virions are self-assembled, allowing them to escape the cell through exocytosis (V’kovski et al., 2021).

## 3 General relationships between SARS-CoV-2 and mitochondria from cellular perspective

The current research showed a wide variety of mitochondrial activities that are severely affected by SARS-CoV-2 infection. Particularly, those findings have firmly established the key role of mitochondria in COVID-19 pathogenesis due to the characterization of (1) subtle interactions between these organelles, the immune system, and mitochondrial damage-related molecular patterns, (2) alterations in mitochondrial functionality and motility, (3) multiple interactions between SARS-CoV-2 proteins and the host mitochondrial proteome, and (4) participation of mitochondria in cellular signaling severely affected by SARS-CoV-2 (Shoraka et al., 2023b). Mitochondrial-mediated antiviral immunity represents the first line of patient defense and mitochondria-related gene expression, mitochondrial functions, and related metabolic pathways are altered in patients with COVID-19 (Duan et al., 2022). Furthermore, accelerated by senescence multiple mitochondrial dysfunctions, including ROS increase, oxidative damage, increased mitogenome mutation rate, OXPHOS deregulation, as well as decrease in ATP production, coenzyme Q (CoQ) level, and failure in antioxidative protection mechanisms, contribute to factors that affect the severity of COVID-19 and lead to increased mortality. The other well-known factors cover age, immunosuppression, hypertension, diabetes, as well as population factors, related to its high density, and anatomical and transmissibility factors (Moreno Fernández-Ayala et al., 2020; Shenoy, 2020; Ganji and Reddy, 2021).

ER membranes were initially shown to be the main centers for the formation of membrane vesicles containing SARS-CoV-2 particles. However, by analogy with other double membrane vesicle (DMV)-inducing viruses, it became evident that mitochondria and various lipid droplets also participate in the biogenesis of SARS-CoV-2 (Roingeard et al., 2022). Some heat shock proteins associated with ER (for example, HSPA5) in ER stress caused by SARS-CoV-2 infection, can move to the cell surface as well as to mitochondrial and nuclear membranes. HSPA5 protein participates in signaling pathways that alleviate SARS-CoV-2 entry and also plays a critical role in the SARS-CoV-2 invasion among cancer patients (Li et al., 2023b). Furthermore, mitochondrial dysfunctions in human diseases, for example, mitochondrial myopathies and neuropathies, ethylmalonic aciduria, Friedreich ataxia, hereditary spastic paraplegia 7, Wilson disease, ageing, cancer, and lung fibrosis, can also affect symptoms and mortality of COVID-19, which also indicates the important role of mitochondria in the development of COVID-19 (Sharma and Sarode, 2022).

From the beginning of the COVID-19 pandemic, mitochondria were suggested to play a critical role in the endocytic pathway that allows SARS-CoV-2 replication and survival in cells, because many viruses have evolved to hijack immunometabolic and mitochondrial functions, resulting in bioenergetic cell deficiencies, dysfunctional mitophagy, Ca^2+^ and ROS imbalance, and other consequences, including preference for aerobic glycolysis to favor SARS-CoV-2 replication (Chapter 5; Nunn et al., 2020; Wang et al., 2020). Mitochondrial response of cell cultures related with the activation of the innate immune and alterations in the protein quality control differs between various coronaviruses (Kohli et al., 2022; Nunn et al., 2022). It was also proposed that mitochondria-endosome interactions are critical for lysosome degradation functions and that mitochondrion may play an important role in SARS-CoV-2 replication (Chapter 8; Swain et al., 2021).

Reactivation of some viruses (e.g., Epstein-Barr [EB] virus) is also associated with severely hijacked mitochondrial functions, such as “long COVID.” “Long COVID” represents post-acute sequelae of COVID-19 (PASC) that can affect at least half of COVID survivors in half of the year after recovery. It is characterized by continuous pulmonary and neurologic symptoms, mental health and mobility impairments, difficulty concentrating, generalized anxiety disorders, fatigue, and muscle weakness, and some multisystemic disorders (Groff et al., 2021). EB virus becomes dormant in a large percentage of the population and was shown to induce the expression of the ACE2 receptor and the entry of SARS-CoV-2 into epithelial cells (Verma et al., 2021). Therefore, it was speculated that EB virus may co-invade COVID-19 patients coincidentally or be truly reactivated due to the impaired immunity of the patient. The association of EB virus infection with mitochondrial aberrations and post-infective fatigue (similar to PASC symptoms), reported by Vernon et al. (2006), could play a key role in the susceptibility and recovery of COVID. This would affect mitochondria in a similar way to SARS-CoV-2 infection; therefore, care should be taken when discussing cellular effects in coronavirus because they can interfere with the effects of EB infection. Among patients mildly suffering from COVID-19, reactivation of the EB virus could further enhance a longer-term secondary syndrome if mitochondrial aberrations are still present (Nunn et al., 2021), indicating the importance of the current physiological status of mitochondria in this process.

Interactions of viral proteins with mitochondria accelerate the overreaction of immunity resulting in permanently increased inflammatory processes, which is one of the ‘long COVID’ features (Yokota et al., 2021; Chen et al., 2023a). One of the more fascinating factors that may affect such a relationship are prion diseases. Stefano et al. (2023a) reported a quite surprising link between the pathophysiological sequelae of “long COVID” and the pathogenesis of prion diseases. Authors proposed a mechanism in which SARS-CoV-2 targets mitochondria and promotes their dysfunction, speculating that ROS burst under SARS-CoV-2 infection can lead to misfolding of prion proteins that can be further accelerated, thus increasing neurodegenerative symptoms in selected tissues, and leading to “long COVID.” In this model “long COVID” with multiple behavioral changes resulting from neuron alterations and neurodegeneration, may arise from prion mobilization and/or SARS-CoV-2 infection. Neurodegeneration that comes from prion misfolding may therefore mask the “true” effects of SARS-CoV-2. According to Stefano et al. (2023a), the pathophysiological effects of “long COVID” may involve the induction of spontaneous production of infectious prion species, especially in individuals susceptible to its origin. The authors concluded that such prion mobilization may explain some “long COVID” symptoms. Recently, aberrant mitochondrial quality control (including dysfunctional mitophagy) has been suggested to contribute to the pathogenesis of numerous diseases, including prion and scrapie infections, that are associated with the an increased pool of intramitochondrial ROS and decreased mitochondrial membrane potential (Kim et al., 2022). However, the unexpected link between prion action, SARS-CoV-2 infection, “long COVID,” and mitochondrial biogenesis needs to be further explored.

In COVID-19, very devastative inflammatory responses accompanied by an increase in the level of pro-inflammatory serum cytokines and chemokines, as well as an increase in neutrophil and macrophage count and the decrease in the endogenous antiviral response from CD8^+^ T cells, NK cells, and gammaDelta T cells (Jaiswal et al., 2020; Potter et al., 2020). Hyperinflammation is induced by the NLR3 pathway by N-protein of SARS-CoV-2, which promotes interactions between NLRP3 and ASC proteins and further results in the IL-1β and IL-6 activation (Pan et al., 2021). Activation of the NLRP3 inflammasome, which contributes to the severity of COVID-19 was studied in *in vitro* infected macrophages and monocytes, in mouse models, and in lung cells with severe COVID-19 (Dutta et al., 2022). Recently, it was suggested that SARS-CoV-2 employs non-canonical activation of NLRP3 mediated by caspase 4/11 (Rodrigues and Zamboni, 2023).

A controlled ROS pool in mitochondria is needed to maintain the continuity of multiple processes and signaling cascades. However, an elevated level of ROS leads to chronic oxidative stress, which can cause cell damage (Giorgi et al., 2018). ROS of mitochondrial origin are important modulators of the inflammatory response in numerous cells, including epithelial cells, and the crosstalk between epithelial and endothelial cells specifically modulates alveolar-capillary injury under COVID-19 (Wang et al., 2020). As chronic state may be initiated by SARS-CoV-2 infection of the epithelium, Chang et al. (2021) proposed that SARS-CoV-2 activates ROS-mediated feedback loops that cause permanent alterations in epithelial redox status and functions, promoting cardiovascular disease and lung injury after COVID-19 recovery. Inflammatory responses lead to the rare event known as the cytokine release syndrome or “cytokine storm,” resulting not only in abnormal coagulation, excessive oxidation, organ damage, immune deficiencies, intravascular coagulation, and organ failure, but also in numerous mitochondrial dysfunctions. Interestingly, pro-inflammatory cytokines induce the activity of indoleamine 2,3-dioxygenase (IDO), which produces kynurenine activating the aryl hydrocarbon receptor (AhR) that modulates mitochondrial metabolism (Anderson et al., 2020). However, its contributions to the COVID-19 pathogenesis still need more studies. In general, the initial phase of pro-inflammatory cytokines inhibits the endogenous antiviral response (Anderson et al., 2020). Cell inflammasome is severely activated by SARS-CoV-2 and mitochondrial dysfunctions accompany chronic inflammation, the “cytokine storm,” inhibited IFN-I release, and the affected immunological responses (Moreno Fernández-Ayala et al., 2020). One of the gatekeepers that regulates mitochondrial-inflammasome interdependencies is the mitochondrial protein NLRX1, a member of the NOD-like receptor family and a potent pattern recognition receptor; it also affects the inflammatory response in COVID-19 (Pickering and Booty, 2021).

The enhanced mitochondrial senescence is accompanied by reprogramming of the immune system, increased oxidative stress, increased mutation ratio in the mitogenome, and increased deregulation of OXPHOS, as well as with a substantial decrease in the pool of naturally occurring antioxidant compounds, as well as CoQ10 and ATP levels. The ultrastructure of aged mitochondria and mitochondria under SARS-CoV-2 infection is also severely affected, e.g., in lung cells and retina; these deficiencies are accelerated by COVID-19, by the appearance of swollen mitochondria, in some cases containing numerous lipid droplets in matrix (Chow et al., 2021; Nardacci et al., 2021; Araujo-Silva et al., 2023). In general, mitochondrial senescence contributes to the malignant prognosis of COVID-19 and to the continuous activation of the inflammasome.

## 4 Aberrations across systems and tissues in COVID-19, and their relevance to mitochondria

“Life cycle” of SARS-CoV-2 can lead to a higher viral load in certain tissues and cell lines. COVID-19 was believed to be primarily associated with serious aberrations in the respiratory system; however, over time, multiple evidence of the devastating role of SARS-CoV-2 in other systems, including the central nervous system was collected (Stefano et al., 2021a; Stefano et al., 2021b; Denaro et al., 2022).

Tissues with slow blood circulation and neurons that require oxygen for OXPHOS activity and ATP synthesis seem to be ideal targets for coronavirus infection, allowing for the high level of replication of SARS-CoV-2, for example, in some brain tissues that exhibit slightly hypoxic conditions. The energy metabolism of neurons may become compromised under SARS-CoV-2 infection, where cells with high oxygen demand become dysfunctional. It was proposed that targeting of neuronal mitochondria by SARS-CoV-2 is selective, induces a “brain fog,” and results in behavioral changes (Stefano et al., 2021b).

The general mechanism of neuropsychiatric manifestations in COVID-19 is very complex; mitochondrial aberrations, leading to systemic downregulation of metabolic activity and cellular bioenergetics within central nervous system cells, were recently investigated (Stefano et al., 2021b; Stefano et al., 2022). ACE2 receptors are also present in neuronal and glial and epithelial cell plasma membranes. The α-cobratoxin domain of S-protein allows interaction with α7 nicotinic acetylcholine receptors (Quiles et al., 2020; Valdés-Aguayo et al., 2021a), however this domain prevents mitochondrial-driven apoptosis if the SARS-CoV-2 replication cycle has not yet finished (Kalashnyk et al., 2021). In dopaminergic brain neurons, ACE2 activity releases angiotensin heptapeptide (Ang-1-7) and alamandine at high levels. Both products bind to the Mas-related gene receptor (Mrg) at the outer mitochondrial membrane (OMM) that is responsible for NO synthesis. Both the abundances of ACE2 and MrgE decrease with age. As ACE2 is bound by S-protein of SARS-CoV-2, ACE2/MrgE/NO signaling pathway that releases NO interferes with coronavirus infection (Valenzuela et al., 2021).

The SARS-CoV-2 sZeta and sGamma variants differentially altered the expression profiles of inflammatory protein genes in rat hippocampus cells exposed to sera containing these variants; moreover, a significant decrease in the expression of mitochondrial biogenesis genes was observed in hippocampi treated with serum samples derived from patients infected with sZeta and sGamma and from individuals with post-COVID syndrome, while a reduction in mitochondrial dynamics was only observed in the latter case (Montenegro et al., 2023).

The effects of coronavirus infection on the nervous system could be seen not only in neurons, but also in the endothelium and microglial cells, and microglia link SARS-CoV-2 infection especially notable with mitochondrial biogenesis, “long COVID” events and chronic inflammation (Stefano et al., 2021a). On the other hand, aberrations of cerebral endothelium respiration are related to mitochondrial damage induced by S1- and Trimer proteins (Kim et al., 2021). When microglial cells were treated with S-protein or inactivated SARS-CoV-2 virions, specific alterations in mitochondrial biogenesis occurred through increased ROS levels, leading to a decrease in mitogenome copies and increased phospholipid saturation (Pliss et al., 2022). Those harmful responses within the central nervous system can be further exacerbated among patients suffering from additional diseases, for example, neurodegenerative ones (Denaro et al., 2022). Since multiple neurological disorders are associated with COVID-19 pathogenesis, damaged mitochondria (with severely affected functionality) and migrasomes (migration-dependent membrane-bound vesicles containing cell content) are present in brain cells infected with SARS-CoV-2; the oxidative stress in brain cells can affect innate immunity leading to impaired cognitive ability (Denaro et al., 2022; Lin et al., 2022; Zhang et al., 2022; Büttiker et al., 2023). The interactome analyses employing viral and neural mitochondrial proteins support these relationships (Maurya et al., 2022). Autopsies from deceased patients indicated a high viral load in the central nervous system, particularly in the brain and Swain et al. (2021) proposed a mechanism of mitochondrial and mTOR dysfunction in infected neurons, where a high level of ROS/Fe accelerates inflammasome hyperactivation and leads to the increased apoptosis. Peluso et al. (2022) proposed that the abundance of SARS-CoV-2 N- and S1-proteins in plasma neuron-derived and astrocyte-derived extracellular vesicles could serve as long-prognostic biomarkers. In particular, SARS-CoV-2 penetrates the nervous system, not only the brain, but also invades enteric neurons and the spinal cord, resulting in cell damage due to inflammation, oxidative stress, mitochondrion dysfunction, and poorly controlled proteostasis (Kaundal et al., 2021).

SARS-CoV-2 infection is related to brain-blood barrier dysfunctions caused by “cytokine storm” and hyperactive immunity, and it also initiates thrombosis in the bloodstream (by activating specific prothrombotic factors). Furthermore, the activity of the brain-blood barrier can be altered among patients with neurodegenerative disorders suffering from SARS-CoV-2 infection, while the physiological state of mitochondria is also negatively altered (Kaundal et al., 2021; Denaro et al., 2022). Recent reports also link the ageing of the blood-brain barrier with increased susceptibility to SARS-CoV-2 infection, which also employ mitochondrial functions (Adesse et al., 2022; Denaro et al., 2022).

Mitochondrial dysfunctions due to SARS-CoV-2 infection alter the functioning of other tissues and organs. Bartolomeo et al. (2022) indicated a preferentially increased susceptibility to SARS-CoV-2 infection in liver, intestine, and breast-derived cell lines, which was connected not only with aberrations in the numerous OXPHOS protein level (including ATP synthase, COX and NDUFS2 subunits), but also with the higher expression level of the TMPRSS2 protein. As mitochondria dominate in hepatocyte metabolism, COVID-induced liver injury (either due to cytopathic effects or due to “the inflammatory burst”) was suggested to be a consequence of mitochondrial dysfunctions in hepatocytes (Morio et al., 2021; Akbari and Taghizadeh-Hesary, 2023). SARS-CoV-2 seriously affects hepatocyte mitochondria, using them for replication and leading to decreased OXPHOS activity (Akbari and Taghizadeh-Hesary, 2023). Multi-organ damage during COVID-19 also affects the kidneys due to acute kidney injury. Mechanisms of kidney injury in COVID-19 comprise systemic immune and inflammatory responses induced by viral infection, systemic hypoxia, reduced renal perfusion, endothelial damage and direct epithelial infections, and mitochondria are key organelles that contribute to the deregulation of the anti-inflammatory response in kidney cells under SARS-CoV-2 infection, where increased glycolysis rate and the reduced oxidation of fatty acids occurred (Kellum et al., 2020).

Placenta belongs to other organs seriously affected by SARS-CoV-2. Mandò et al. (2021) reported multiple aberrations in placenta cells driven by SARS-CoV-2 presence which covered various alterations in the mitochondrial biogenesis including decrease in the mitogenome copy number and the antioxidant protein level. The downregulation of the OXPHOS protein genes (e.g., *NDUFA9*, *SDHA*, *COX4I1*) and the motility genes (e.g., *DNM1L*, *FIS1*) was also notable. These effects were attributed to the worsening intrauterine environment, leading to the deepening of placental oxidative stress and the decrease in mitochondrial performance. Additionally, Liu et al. (2022) noted a general increase in abnormal mitochondria and an elevated mitogenome content in placentas of mothers infected with SARS-CoV-2, which was speculated to be associated with transient early fine motor abnormalities among newborns. Gabanella et al. (2022) also studied the effects of SARS-CoV-2 in trophoblasts and found the presence of the coronavirus genome within host mitochondria, which was associated with global reprogramming of mitochondrial net and mitochondrial activities. Furthermore, Sun et al. (2022) speculated that SARS-CoV-2 may possibly also affect female fertility by hijacking mitochondrial activity.

## 5 SARS-CoV-2 sensing through mitochondria- from MAVS to respiratory aberrations, OXPHOS deficiencies and perturbations in iron homeostasis

**FIGURE 3:**
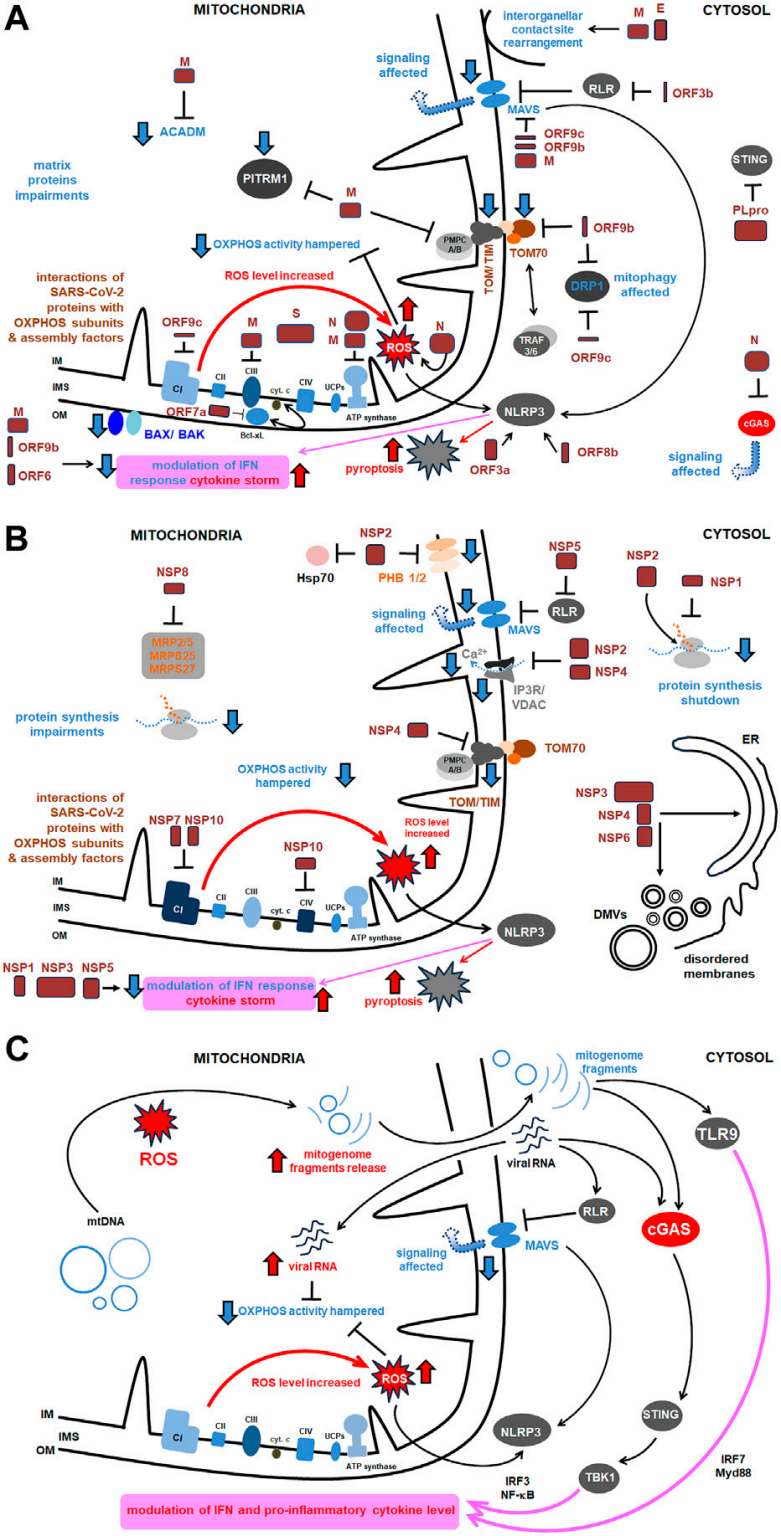
The comprehensive scheme of interactions of SARS-CoV-2 with host mitochondrial proteins and diverse mitochondrial activities. Key proteins as well as the most relevant pathways affected under SARS-CoV-2 infection were shown. More details in the text. **(A)** Main interactions between structural proteins of SARS-CoV-2 and host mitochondrial proteins and activities; **(B)** Main interactions between virus NSPs and mitochondrial proteins and activities (the remaining ones are presented in Table 1); **(C)** Main signaling routes for SARS-CoV-2 RNA and the released mitogenome fragments in the host cell. ACADM, acyl CoA dehydrogenase medium chain; Bax/Bak, Bcl 2-like protein 4/BCL2 antagonist/killer 1; Bcl xL, B cell lymphoma extra large; CI, CIV, respiratory chain complexes; cGAS, cyclic GMP–AMP synthase; COX, cytochrome *c* oxidase; cyt., cytochrome; DMVs, double membrane vesicles; DRP, dynamin related protein; E, SARS-CoV-2 envelope protein; Hsp, heat shock protein; IFN, interferon; IM, inner membrane; IMS, intermembrane space; IP3R, inositol triphosphate receptor; IRF, interferon release factor; MAVS, mitochondrial antiviral signaling; MRP, multidrug response protein; MTP, mitochondrial peptidase; Myd, myeloid differentiation primary response; N, nucleocapsid protein; NF-κB, nuclear factor kappa-light- chain-enhancer of activated B cells; NLRP, nucleotide-binding oligomerization domain; leucine rich repeat and pyrin domain containing; NSP, non-structural protein; OM, outer membrane; ORF, open reading frame; OXPHOS, oxidative phosphorylation; PITRM1, pitrilysin metallopeptidase; PHB, prohibitin; PLpro, polyprotein; RLR, RIG-I-like receptor; ROS, reactive oxygen species; S, ‘spike’ protein; STING, stimulator of interferon genes; TBK, TANK binding kinase; TIM, translocase of the inner membrane; TLR, Toll-like receptor; TOM, translocase of the outer membrane; TRAF, tumor necrosis factor receptor–associated factor; UCP, uncoupling protein; VDAC, voltage dependent anion channel.

The presence of SARS-CoV-2 RNA is detected by the RIG-I receptor (retinoic acid-inducible gene I) receptor, which activates MAVS (mitochondrial antiviral signaling protein), a part of the mitochondrial signalosome complex located in OMM (Figure 3). MAVS affects the level of antiviral IFN that regulates viral replication by interaction with a viral inhibitor (viperine; Kloc et al., 2020). Recently, numerous SARS-CoV-2 proteins have been shown to facilitate MAVS degradation, e.g., ORF10. Under overexpression, it participates in ORF10-induced autophagy that includes the degradation of the mitochondrial signalosome. ORF10 can also induce mitophagy through interaction with the Nip3-like protein X (NIX) mitophagy receptor, which can also subject MAVS to degradation. Through these activities, ORF10 facilitates SARS-CoV-2 replication (Li et al., 2022).

SARS-CoV-2 infection affects the expression of several cellular genes. Perturbations in mitochondrial activity, inflammation, and autophagy pathways were specifically observed for SARS-CoV-2 infected cells; they were associated with downregulation of mechanistic target of rapamycin (mTOR) components, as well as mitochondrial ribosomal and mitochondrial complex I (CI) subunits, and proteins for lysosome acidification (Singh et al., 2020b). Using single cell RNA-seq data from human bronchial epithelial cells, colon and ileum organoids, Gutiérrez and Elena (2022) showed distinctness of expression profiles for a variety of mitochondrial genes under SARS-CoV-2 infection. SARS-CoV-2 affects mitochondrial respiratory metabolism, switching from OXPHOS activity to glycolysis, and, in general, it indirectly alters the immune system response (Kloc et al., 2020). SARS-CoV-2 leads to downregulation of numerous nuclear OXPHOS genes, especially those coding CI subunits (Figure 3). SARS-CoV-2 can affect the expression level of mitochondrial protein genes as early as 2 h after infection (Miller et al., 2021; Archer et al., 2022). As most of the mitochondrial proteome is encoded by nuclear genes, they are particularly contributing to the pathogenesis of COVID-19.

Carotid bodies allow for oxygen detection and proper chemoreflex, and decreased OXPHOS and MAVS activities, together with ROS burst, severely alter their functions, leading to hypoxemia. However, aberrations in mitochondrial biogenesis affect damage in the respiratory system, where not only oxygen sensing, but also pulmonary artery constriction is impaired and vascular damage progresses (Ackermann et al., 2020; Archer et al., 2020; Gattinoni et al., 2020). These responses are accompanied by a decrease in the IF level and an increase in the level of oxidized biomolecules and a severe inflammation (Burtscher et al., 2020). In the human nasal epithelium, Yeung-Luk et al. (2023) investigated tissue remodeling, which includes relatively early aberrations in mitochondrial activity, decreases in oxygen consumption and extracellular pH, and increases in actin polymerization that led to general remodeling of the cytoskeleton.

Mitochondrial dysfunctions were also studied in peripheral blood monocytes from COVID-19 patients. ATP synthesis, respiratory rate in state 3, basal respiration rate, and maximal respiratory capacity in these cells were all decreased (Ajaz et al., 2020; Gibellini et al., 2020). Additionally, SARS-CoV-2 led to the accumulation of advanced glycation end products. Interestingly, the proton leak across the mitochondrial membrane was also less effective. Gibellini et al. (2020) also reported the subsequent increase in tumor necrosis factor (TNF) and IFN synthesis, and the increase in mitochondrial size under COVID-19 progression, which appeared slightly less round and larger. According to Gibellini et al. (2020) results, mitochondria of monocytes from COVID-19 patients were heterogenous in size, swelled, and with electron-lucent matrix. They had increased both area, as well as external perimeter and Feret’s diameter by 4.3, 1.2 and 1.2 times, respectively. The roundness of these mitochondria significantly decreased, however, the aspect ratio increased almost by 1.1 times. SARS-CoV-2 infection of the lung epithelial cell line increased pyruvate kinase muscle isoform 2 (PKM2); however, the PKM2-specific stabilizer restored glycolytic aberrations (Allen et al., 2022). HIF-1α (hypoxia inducible factor 1α) together with the ORF3a protein, plays an important role during SARS-CoV-2 infection and the development of the pro-inflammatory response (Figure 3) (Tian et al., 2021) and induce the expression of glycolytic genes. Finally, the energetic deficit and the glycolytic switch increase the pro-inflammatory response, decrease the level of IFN and the rate of lymphocyte T proliferation, and severe COVID-19 symptoms; the higher glucose level favors SARS-CoV-2 infection with vast glycolysis (Codo et al., 2020). The level of interleukin-6 (IL-6) increased in blood mononuclear cells among both SARS-CoV-2 infected and deceased patients (Ajaz et al., 2020). Among severely affected COVID-19 patients Romão et al. (2022) noted that the elevated SARS-CoV-2 load induced an increase in mitochondrial ROS level associated with the altered pattern of CD14^+^ monocytes, with decreased mitochondrial membrane polarization.

In general, the studies discussed above support the relevance of monocytes in the pathogenesis of COVID-19. Generally, in leukocytes, under SARS-CoV-2 infection, inflammation and oxidative damage are associated with affected mitochondrial functions and increased probability of apoptosis; these replies explain the rapid changes, that overactivated the immune system (De la Cruz-Enríquez et al., 2021). Regarding other blood elements, human platelets (often enlarged) in COVID-19 patients contain hyperpolarized mitochondria and significantly reduced intracellular Ca^2+^ pool, however expression profiles of OXPHOS genes are not affected, despite permanent inflammation (Yasseen et al., 2022).

The level of OXPHOS proteins in neural cells is also markedly reduced in some cases of COVID-19. Peluso et al. (2022) noted that the lower abundance of CI subunit 6, CIII subunit 10, VDAC1 and the neuroprotective humanin was associated with PASC with neuropsychiatric manifestations. Emerging evidence is collected to support the idea that disruption of NAD^+^ metabolism and subsequent mitochondrial dysfunctions accompanying the integration of the SARS-CoV-2 genome may, in fact, contribute to the pathogenesis of post-acute sequelae of SARS-CoV-2 (Block and Kuo, 2022).

Mitochondria also belong to key organelles involved in cellular iron (Fe) turnover. This element is present within these organelles among the well-known hem residues, Fe-S clusters, and Fe-binding proteins, including ferritin. In severe COVID-19, an increased iron level was detected in hepatocytes among patients with liver injury during SARS-COV-2 infection; therefore, it was suggested that hepatic iron overload could enhance liver injury associated with COVID-19 (Del Nonno et al., 2021). The increased level of free iron within mitochondria is a consequence of pro-inflammatory processes, and its overaccumulation promotes further oxidative stress, leading to lipid peroxidation and decreased glucose tolerance. Mitochondria are sensitive to oxidative stress under elevated iron level, which is often a consequence of high ferritin level (hyperferritinemia) and is associated with severe outcomes of COVID-19 (Saleh et al., 2020; Mahroum et al., 2022). Deepened oxidative stress leads to ferroptosis, mitochondrial dysfunctions, and finally organ damage. Interestingly, some microbiota and platelet dysfunctions (with depolarized mitochondria and other organellar aberrations) could be enhanced by mitochondrial dysfunctions that also progress them, leading to hypercoagulopathy, reported during COVID-19 progression. Surendran et al. (2022) observed spatiotemporal deregulation of the mitochondrial isoform of heme oxygenase 1 (HMOX1), proving the relevance of Fe metabolism in the pathogenesis of COVID-19. Lipid peroxidation, resulting from the iron release from Fe-storing proteins, is induced by tissue acidosis and intramitochondrial ROS burst in COVID-19 and leads to further mitochondrial damage. Jovandaric et al. (2022), based on recent reports, speculated that such mitochondrial aberrations could result in increased mortality of newborns and pregnant women under SARS-CoV-2 infection. Furthermore, in COVID-19, in addition to the increase in intramitochondrial iron level, the distribution of Mn is also altered, which affects Mn-superoxide dismutase (Mn-SOD) activity (Denorme et al., 2020; Edeas et al., 2020; Saleh et al., 2020).

## 6 Complexity of SARS-CoV-2 protein interactions and activity

Flynn et al. (2021) have analyzed the SARS-CoV-2 interactome, which consists of multiple cellular proteins. The most abundant families comprised RNA-binding proteins, heterogenous ribonucleoprotein particles (hnRNPs), the translation apparatus, metabolic enzymes, cytoskeleton components, and intracellular vesicles protein components. However, most SARS-CoV-2 proteins also interact with host mitochondrial proteins (Figure 3). The interaction of SARS-CoV-2 with mitochondria allows the initial opening of the mitochondrial permeability transition pore, mitochondrial membrane depolarization, and the ROS release (Shang et al., 2022). Some of the mentioned below proteins affect mitochondria, including antiviral signaling, mitochondrial motility, ion homeostasis, as well as expression profiles of genes for various mitochondrial proteins; all that illustrates complexity of their action.

One of the best-known interactions between coronavirus and host cell is driven by S-protein. It is responsible for viral transmission and pathogenesis and modulates the host immune response. S-protein forms a complex composed of two proteins of (S1/S2)_3_ stoichiometry, with the surface subunit S1 and the S2 protein interacting directly with the membrane. S-protein precursor is hydrolyzed by a furin-like protease, resulting in the formation of mature S1 and S2 subunits. The S1 subunit contains an N-terminal domain (NTD) and a receptor binding domain (RBD). In contrast, the S2 subunit has a fusion peptide (FP), heptad repeat 1 (HR1), heptad repeat 2 (HR2), transmembrane domain (TM), as well as a cytoplasmic tail at the C end (CT) (Figure 1) (Ghimire et al., 2022). S-protein binds the opportunistic angiotensin-converting enzyme-2 receptor (ACE-2) and the transmembrane serine protease (TMPRSS2) to the cell plasma membrane. This interaction is followed by internalization of S1 RBD and the formation of active trimer of S-protein. Endosomal vesicles are generated by membrane fusion, which allows viral entry by endocytosis (Kim et al., 2021). However, an alternative pathway for virus penetration was proposed. For example, ACE2/“spike”-independent infection of T lymphocytes is probably mediated by the lymphocyte function-associated antigen (LFA-1) expressed in these cells (Shen et al., 2022).

According to Zekri-Nechar et al. (2022), S-protein subunits affected mitochondrial membrane potential, decreased respiratory rate, and increased mitochondrial fission rate in human pulmonary microvascular endothelial cells (HPMEC). Cao et al. (2023) showed that S-protein induces long-term transcriptional suppression of mitochondrial metabolic genes (including ATP synthase and CI subunit genes) and upregulates genes for factors related to the stress pathway and genes for glucose metabolism. Consequently, cardiac impairments (including cardiac fibrosis) develop in obese mice with PASC; moreover, lipid and obesity cause selective accumulation of SARS-CoV-2 in heart, aorta, and adipose tissues. In general, ROS and intramitochondrial Ca^2+^ levels increased in human cardiomyocytes under SARS-CoV-2 infection (Huynh et al., 2023). Furthermore, Lesiów et al. (2023) showed that peptides derived from S-protein together with Cu^2+^ synergistically induce an increase in intramitochondrial ROS. This finding highlights the relevance of interactions between SARS-CoV-2 proteins with metals, which contribute to lung damage in COVID-19.

M-protein, which is the most abundant of the transmembrane proteins, is associated with the morphology of SARS-CoV-2 and key steps of its replication. Yang et al. (2022) suggested that the structural M-protein of SARS-CoV-2 exhibits pro-apoptotic properties in the lung epithelium and causes lung edema. M-protein stabilizes the B cell lymphoma 2 ovarian killer (BOK) by inhibiting its degradation and promotes the translocation of BOK mitochondria. Furthermore, M-protein negatively affects mitochondrial MAVS aggregation and signaling (Fu et al., 2021) and downregulates the IFNβ and IFN-stimulated gene expression (Sui et al., 2021). It also interacts with the ATPase Na^+^/K^+^ transporting subunit β1 (ATP1B1), the vacuolar ATPase H^+^ transporting V1 subunit A (ATP6V1A), acyl-CoA dehydrogenase (ACADM), ALADIN protein, mitochondrial peptidase processing subunits (PMPCA, PMPCB), pitrilysin metallopeptidase (PITRM1) and the coenzyme Q8B (COQ8B) (Singh et al., 2020b).

E-protein, a conserved viroporin, is involved in the release of viral particles from cells and modulation of the activity of membrane ion channels. Together with M-protein, it regulates intracellular trafficking of S-protein as well as its intracellular processing (Boson et al., 2021), and affects Ca^2+^ homeostasis, and participates in the rearrangement of interorganellar contact sites, leading to mitochondrial remodeling (Poggio et al., 2023).

N-protein affects viral replication, transcription, and assembly together with SARS-CoV-2 NSPs and suppress host cell apoptosis by interaction with MCL-1 anti-apoptotic protein; this may explain SARS-CoV-2 effective replication in asymptomatic patients without respiratory dysfunction (Hirabara et al., 2022; Pan et al., 2023). N-protein also disrupts the assembly of cGAS with its co-factor G3BP1, which reduces IFN-I signaling (Cai et al., 2023). This reply opposes the accumulation of mitogenome fragments that activate the cGAS pathway (Chapter 7). N-protein also increases the mitochondrial ROS level, affects ATP synthesis, increases CI and CIII activity and upregulates the expression level of mitochondrial genes for their subunits; it stabilizes mitochondrial transcriptional complexes and thus belongs to SARS-CoV-2 proteins seriously affecting host mitochondria (Yu et al., 2023).

Other SARS-CoV-2 proteins, including ORF3a, ORF9c, and ORF10 bind to various mitochondrial proteins, including components of the mitochondrial PTP complex, cyclophilin D, SPG-7 paraplegin, ANT transporter, ATP synthase, and uncharacterized CCDC58 protein (Figure 3) (Ramachandran et al., 2022). ORF3a, a monomer or dimer protein on the plasma membrane, which is synthesized in the ER, recruits some host proteins that induce mitochondrial damage and ROS production. This facilitates HIF-1α production and obviously accelerates further ROS production, release of mitogenome fragments by NIM811-sensitive mitochondrial-permeability-pore (Chapter 7) and NLRP3 inflammasome. ORF3a also interferes with proteins that regulate mitochondrial fission (Tian et al., 2021; Guarnieri et al., 2023; Jiao et al., 2023). ORF3b protein interacts with the RIG-I-like receptor (RLR), which also affects MAVS signaling. ORF3c targets mitochondria and evades host immunity by restricting IFN-β production, however, not by changing JAK-STAT signaling (Stewart et al., 2023). ORF7a interacts with Bcl-XL, thus affecting programmed cell death (PCD). The proteins ORF3a and ORF8b interact with NLP3, which regulates the pro-inflammatory cascade and pyroptosis (Figure 3).

ORF9b protein, together with ORF6, inhibits, by the direct action, IFN signaling within the infected cell. ORF9b inhibits the activities of the poly (rC) binding protein (PCBP2) and the E3 ligase of the HECT domain (AID4E3) necessary for the activation of the MAVS signalosome, which affects mitochondrial anti-viral signaling (Malavolta et al., 2020). ORF9b protein also induces the formation of membrane vesicles containing mitogenome fragments; moreover, it causes extensive mitochondrial remodeling (Chapter 9) and, together with NSP4, forms OMM macropores, which influences mitochondrial motility and ultrastructure (Faizan et al., 2022). ORF9c protein became another object of the studies. It directly inhibits the MAVS signalosome and the Drp1 protein (which promotes mitochondrial elongation over fission) and ORF9c binds to the NDUFAF1 and NDUFB9 proteins, altering the functioning of OXPHOS. Consequently, pro-inflammatory factors are released, neutrophiles and monocytes are hyperactivated, antiviral signaling, and host kinase activities are altered, OXPHOS gene expression is severely downregulated and mitochondrial senescence is enhanced (Figure 3; Malavolta et al., 2020).

Regarding the activity of various NSPs, NSP1, NSP2, NSP3, and NSP5 are particularly important for viral biogenesis. NSP1, which belongs to the initial synthesized SARS-CoV-2 proteins in the host cell, participates in translational shutdown (inhibits host protein synthesis) and host mRNA decay only after ribosome engagement with the transcript (Alanagreh et al., 2020; Shehata and Parker, 2023). NSP1 through inhibition of translation and induction of mRNA degradation targets the translated transcript pool. The N terminal part of NSP1 stabilizes the binding of the C-terminus and nonspecifically blocks the mRNA channel in the 40S ribosomal subunit, suppressing host gene expression. Interestingly, the fragment of the natively unstructured C-terminus of NSP1 adopts a secondary structure upon binding of ribosomes and is capable of Cu^2+^ binding. Targeting stem-loop 1 of the 5′UTR of SARS-CoV-2 can accelerate NSP1 activity by switching cells to coronavirus protein translation (Mendez et al., 2021; Zhao et al., 2021; Morales et al., 2022; Vora et al., 2022). NSP1, in general, suppresses IFN signaling (Thoms et al., 2020; Fisher et al., 2022).

NSP2 have been shown to hamper intracellular cell signaling by interaction with two prohibitin isoforms (PHB1 and PHB2); it was initially speculated that NSP2 may be involved in altering the host cell environment (Cornillez-Ty et al., 2009). According to Graham et al. (2005), in SARS-CoV-1 this protein is not necessary for infection. However, in SARS-CoV-2, NSP2 enables the “life cycle” of the virus and impairs IFN production by binding to the GIGYF2 protein, which is a translation repressor (through interaction with the cap binding complex) of *Ifnb1* transcripts (Xu et al., 2022). Furthermore, Naeli et al. (2023) showed that NSP2 can also increase miRNA-mediated silencing of cellular transcripts. However, the exact role of NSP2 in translation is still unclear. On the contrary, the Korneeva et al. (2023) report states that translation increased even in the HEK293T cell line that overexpressed NSP2 both under hypoxia and normal growth conditions. NSP2 also affects VDAC2 biogenesis and increases the abundance of stomatin-like 2 (STOML2), which subsequently decreases apoptosis. Together with NSP4, NSP2 affects mitochondrial Ca^2+^ homeostasis (see below). Davies et al. (2020) revealed sets of commonly and uniquely interacting partners of NSP2 proteins from SARS-CoV-2. The SARS-CoV-2 interactome comprised proteins indispensable for mitochondrial biogenesis, including PHBs, VDAC2 isoform and STOML2 protein. Furthermore, the Hsp70 chaperone system participated in interactions with NSP2/NSP4 proteins, as well as with components of the inositol triphosphate receptor (IP3R) ubiquitination system (see below) and proteins indispensable for vacuolar ATPase biogenesis.

In addition to affecting the host’s innate immune response, the NSP3 protein (known also as PLpro, papain-like protease) plays a role in polyprotein cleavage and is a component of SARS-CoV-2 replication transcription complexes (Khan et al., 2021; Ju et al., 2023). Furthermore, PLpro also cleaves host ubiquitin and ISG15 ubiquitin-like protein modifications affecting innate immunity (Calleja et al., 2022). NSP3 and N-protein interactions are essential for the processing of SARS-CoV-2 genomic RNA as well as SARS-CoV-2 fitness and virulence. At least 14 amino acid residues of NSP3 were suggested to be indispensable for virus RNA synthesis (Khan et al., 2021; Li et al., 2023a). Shi et al. (2021) showed that NSP3 exhibits dual subcellular localization (in ER and mitochondria).

NSP4 transmembrane glycoprotein forms DMVs associated with replication complexes (Gadlage et al., 2010). Furthermore, NSP4 (together with NSP2) through the recruitment of the ERLIN1/2 and RNF170 proteins (present in the ER membranes and close to the contact sites) severely affect mitochondrial Ca^2+^ homeostasis at the ER-mitochondrial junctions (Figure 3). These proteins generally block Ca^2+^ efflux through the IP3R receptor from the ER to mitochondria by interaction of the ERLIN1/2 with the E3 ligase and targeting of the IP3R receptor to polyubiquitination, after its interaction with RNF170, which has fatal consequences for Ca^2+^ transport (Davies et al., 2020). In cardiomyocytes infected by SARS-CoV-2, mitochondrial Ca^2+^ homeostasis was severely altered by its cycling alterations (Ramachandran et al., 2022). According to these findings, the level of mitochondrial Ca^2+^ uniporter in neurons was decreased in “long COVID” (Peluso et al., 2022). Furthermore, NSP4 also affects TIM functioning (leading to import impairments) and regulates the level of the negative regulator of cell proliferation. Both NSP4 and ORF9c proteins belong to key coronavirus proteins that affect numerous cellular processes. In particular, the expression of MERS-CoV *nsp3* and *nsp4* genes, as well as SARS-CoV-2, was necessary to form double membrane vesicles (DMVs; Oudshoorn et al., 2017). According to Angelini et al. (2013), overexpression of NSP3, NSP4, and NSP6 in tissue cultures also resulted in DMV and disordered membrane appearance (Chapter 6).

NSP5 (also known as Mpro, main protease for processing SARS-CoV-2 polyproteins) together with N-protein of SARS-CoV-2 block formation of “stress granules” and affect RIG-I signaling by disrupting RIG-I-MAVS complex (Zheng et al., 2022; Ju et al., 2023). In addition, it activates NF-κB signaling pathway through specific SUMOylation of the MAVS signalosome, which increases its stability and further triggers NF-κB pathway (Li et al., 2021). NSP5 targets RIG-I and MAVS in two distinct ways: it cleaves off the 10 most N-terminal residues of RIG-I and deprives it of the ability to activate MAVS and promotes proteosome-mediated degradation of MAVS (Liu et al., 2021). NSP5 also cleaves the melanoma-associated antigen D2 (MAGED2), ubiquitously expressed in host cells and directed at the cytoplasm and nucleus (Ju et al., 2023). In general, NSP5 is responsible for blocking the host’s innate immune response and IFN synthesis.

NSP6 by the oligomerization and an amphipathic helix, zippers membranes of ER and establishes the connectors, as a consequence to the activity of NSP3 and NSP4 generating DMVs. Notably, NSP6 (1) acts as a filter in communication between the replication organelle and the ER; (2) participates in positioning and organization of DMVs; (3) mediates contact with lipid droplets (Ricciardi et al., 2022).

Other non-structural proteins, including NSP7 and NSP10, bind to various proteins for the biogenesis of the OXPHOS machinery (NDUFAF2, NADH4L, COXII36), and NSP8 interacts with various multidrug resistance proteins (MRPs). NSP8, which contains both the mitochondrial target domain and the autophagy/mitophagy-inducing domain, causes an increase in autophagosome accumulation and its action results in incomplete mitophagy (Figure 3) (Zong et al., 2023). NSPs also interact with MAM proteins, which affects the number of contact sites between mitochondria, their motility, and the frequency of apoptosis (Davies et al., 2020).

## 7 Participation of mitogenome fragments in signaling pathways and the variety of COVID-19 mitobiomarkers

Recent studies have shown that the human mitogenome plays a specific role in the pathogenesis of COVID-19. Some mitochondrial gene mutations are uniquely associated with severe cases of COVID-19. Kumari et al. (2023) found at least 15 non-silent mutations in the *ND5*, *ND4*, *ND2,* and *COI* genes. Furthermore, mitogenome haplogroups M3d1a and W3a1b could be particularly associated with the pathogenesis of COVID-19. Mutations within the *ND1* and *ND2* genes and the D-loop were proposed to be related to the increased risk of a severe prognosis of COVID-19 and other mutations in those genes, as well as in the *ND3* and *COI* genes, were associated with the lower risk of such a prognosis among representatives of the Han population (Wu et al., 2021).

The host mitogenome, when released into the cytoplasm, interacts with different receptors, triggering a pro-inflammatory response and activating IFN-I signaling (Figure 3; West, 2017). The endosomal TLR9 (Toll-like 9) receptor recognizes DNA fragments and unmethylated CpG dinucleotides in DNA and enhances Myd88 signaling pathway. Pro-inflammatory cytokine activation can also be regulated by cyclic GMP–AMP synthase (cGAS), which activates STING protein in the ER, which is necessary for the activation of the IRF responsible for the IFN production. Therefore, the released mitogenome fragments could enhance IFN-I signaling through cGAS activity. Next, the cytoplasmic AIM2 factor, which interacts with both DNA grooves, stimulates the activation of the inflammasome. Some pro-inflammatory cytokines also accelerate mitogenome release (Targhetta et al., 2021).

Guarnieri et al. (2023) recently revealed that mitogenome fragment release can be enhanced by the increased abundance of mitochondrial ROS that interact with the NIM811 cyclosporin-sensitive mitochondrial permeability pore (mtPTP), which can further activate the inflammatory response. Mitochondrial dysfunctions during infection of human umbilical vein endothelial cells (HUVAC) by SARS-CoV-2 also included the release of mitogenome fragments, which sensed the vascular reactivity of endothelin-1 in wild-type mice (Costa et al., 2022). In addition, some variations in the copy number of the heteroplasmic mitogenome have been speculated to have functional consequences for immunity within the nervous system (Stefano and Kream, 2022).

The abundance of released mitogenome fragments differs between patients with various manifestations of COVID-19 (Valdés-Aguayo et al., 2021b; Mahmoodpoor et al., 2023). The relationship between the level of cell-free mitogenome fragments was investigated primarily among patients with severe cases of COVID-19. Shoraka et al. (2023a) compared a few copies of the cell-free mitogenome, as well as the abundance of mitochondrial transcription factor A (TFAM) between patients with symptomatic and asymptomatic COVID-19. The abundance of extracellular mitogenome fragments was elevated among asymptomatic patients, and both cohorts did not differ with the abundance of TFAM. Therefore, the authors indicated the importance of mitogenome fragments in the pathogenesis of COVID-19, due to their participation in inflammatory processes. In severe cases of the prognosis of COVID-19, numerous mitogenome fragments were detected in plasma from patients; for example, *CYTB* gene copy number was increased in deceased patients than among those who survived infection. Furthermore, these responses were highly associated with the level of other diagnostic markers, including ferritin, D-dimer, lactate dehydrogenase (LDH), and IL-6 (Scozzi et al., 2021; Valdés-Aguayo et al., 2021a).

Among the mitobiomarkers associated with the pulmonary version of COVID-19 are PTEN-induced kinase 1 (PINK1), dynamin-1-like protein (DNM1L) and mitofusin-2, which supports the relevance of oxidative stress and aberrations in mitochondrial motility (Chapter 9) in COVID-19 pathogenesis. Some other diagnostic proteomic markers that were recently proposed included mitochondrial ribosome large subunit protein (MRPL), L-2-hydroxyglutarate dehydrogenase (L2HGDH), ATP synthase subunits, cytochrome *b* (CYTB), complex I subunit (NDUF) and other mitochondrial proteins (Chen et al., 2023b; Siekacz et al., 2023). The decrease in the level of other mitochondrial proteins, including MOTS-c peptide, VDAC-1 and humanin, was also reported in extracellular vesicles isolated from blood of patients with severe COVID-19. Those proteins were proposed as biomarkers allowing to distinguish between distinct manifestations of COVID-19 (Goetzl et al., 2023). In addition, the mitochondrial mass of circulating NK cells strongly polarized among deceased patients was recently suggested as another marker related to the COVID-19 severity (Wang et al., 2023).

## 8 Subgenomic RNA of SARS-CoV-2 in membrane continuum and the regulation of mitochondrial biogenesis by ncRNA in COVID-19

The cell membrane continuum belongs to the main targets of SARS-CoV-2 and subgenomic RNA (sgRNA) fragments are frequently detected within the lumen of the membrane cisternae within the cell, including organelles (Gatti et al., 2020; Quiles et al., 2020; Wu et al., 2020). Mitochondria belong to highly enriched organelles in the replication products of the SARS-CoV-2 genome. It was speculated that the penetration of dsRNA into mitochondria may be governed by TOM20 activity (Shang et al., 2022). The intramitochondrial localization of the SARS-CoV-2 genome fragments may indicate horizontal gene transfer between the viral genome and mitochondria (Stefano et al., 2023b). Wu et al. (2020) used the RNA-GPS model and advanced *in silico* analyses to predict the probability of the presence of various parts of viral RNA (including 5′ leader and 3′UTR sequences, as well as all coding regions) in the ER membrane, nuclear lamina, mitochondrial matrix and OMM, as well as in the cytosol, nucleolus, nuclear envelope, and the entire nucleus. The highest rank values (vs localized human transcripts) were achieved for sgRNA fragments enriched in the mitochondrial matrix and nucleolus, that included *orf3a*, *e*, *m*, *orf6*, *orf7a*, *orf7b*, *orf8*, *n* and *orf10* (the latter only in the mitochondria).

The level of selected non-coding RNA (ncRNA) appears to be associated with alterations in mitochondrial biogenesis during SARS-CoV-2 infection. A specific microRNAs indispensable for the regulation of gene expression related to mitochondrial biogenesis are known as mitomiRs (Iessi et al., 2021). Recently, miR-195 was shown to discriminate between mild and severe cases of COVID-19. This plasma-isolated miRNA belongs to established responders to viral infections. Moatar et al. (2023) speculated that cardiac mitochondrial homeostasis can be affected in COVID-19 by miR-195, due to the fact that its targets comprise a plethora of mitochondrial biogenesis genes.

Pozzi (2022) explored the impact of SARS-CoV-2 infection on the expression of various mitochondrial ncRNA, despite the small cohort number and ncRNA extracted only from peripheral blood monocytes. The analyzed data (Bioproject NCBI data accession no. PRJNA662985, 2024) came from 85 samples from 18 patients hospitalized between January and April 2020. Short and long RNA expression levels were tested. According to Pozzi (2022), the level of long ncRNAs was not altered in any investigation of SARS-CoV-2 infection stages; instead, up to 43 small mitochondrial RNAs have altered their expression profiles during recovery from COVID-19. The results suggested that, although the general transcription profile of mitochondrial genes was not greatly affected (only expression level of the *ND6* gene for the CI subunit was altered), SARS-CoV-2 infection affected the levels of small organellar RNA. For example, differentially expressed in the clinical stages investigated, due to the independent influence of SAR-CoV-2 on the expression pattern of diverse classes of mtRNAs. Interestingly, the lower abundance of some mtRNAs lasted during post-COVID patient rehabilitation. In addition to that, the study by Ito et al. (2022) revealed a specifically enhanced level of mitochondrial-related transcripts among COVID-19 patients.

## 9 Mitochondrial dynamics in COVID-19

Mitochondria belong to highly polymorphic organelles, with variable size, shape, and interactions at various developmental stages and environmental stimuli. The mitochondrial network behaves as a dynamic grid of tubular or branch-like structure closely related to the frequency of mitochondrial fusion and fission (Logan, 2010; Rafelski, 2013). Diverse patterns of animal mitochondrial fusion/fission involve microfusion (a rare scenario), mesofusion (when fission predominates), dynamic hyperfusion (when fusion predominates), and the rare static hyperfusion (Hoitzing et al., 2015). The mitochondrial net may be composed of both “healthy” and damaged organelles. Long-term stress and viral infections could affect this balance, leading to the appearance of hyperfused mitochondria and mitophagy governed by Parkin, PINK1 proteins, and the TOM/TIM major importin complex. Under physiological conditions, the mitochondrial network allows increased cell proliferation and energy production, regulation of signaling pathways, and anti-apoptotic protection (Hoitzing et al., 2015). Mitofusins belong to main components of the OMM fusion machinery. However, the protein required to perform the integration of the inner mitochondrial membrane (IMM) is the OPA1 protein, the mitochondrial dynamin-like GTPase. In turn, dynamin-related protein 1 (Drp1), fission protein Fis1 and mitochondrial fission factor (Mff) participate in mammalian mitochondrial fission (Westermann, 2012; Gatti et al., 2020; Elesela and Lukacs, 2021).

SARS-CoV-2 infection destroys the mitochondrial quality system. In COVID-19, mitophagy is inhibited at an early stage, despite that PINK1 and the Parkin protein are still recruited (Elesela and Lukacs, 2021; Shang et al., 2022). Additionally, the Mfn2 level increases, leading to remodeling of the mitochondrial network (Motta et al., 2023). During SARS-CoV-2 infection, mitochondrial fission prevails over fusion events, because the activation and massive upregulation of Drp1 GTPase occurs (Archer et al., 2022). For instance, patients with infected alveolar epithelial cells display more frequent mitochondrial fission, inhibited mitochondrial degradation, and one of the Ca^2+^ transporter affected; moreover, after phagocytosis of damaged cells, mitochondrial respiratory capacity decreased and the ROS-HIF1α pathway was activated in macrophages, accelerating the lung inflammatory response (Duan et al., 2022).

COVID-19 also alters mitochondrial morphology and ultrastructure in a direct or indirect manner (Barreto-Vieira et al., 2022). Castro-Sepulveda et al. (2022) showed that the low density of mitochondrial cristae together with their abnormal morphological pattern (shorter cristae), possibly due to physical inactivity, is associated with the severity of COVID-19 symptoms. Interestingly, only patients with mild COVID-19 (but not those with severe cases) had a lower abundance of various mitochondrial proteins, including Opa1, Mfn2 and SDHB (succinate dehydrogenase subunit).

SARS-CoV-2 promotes ORF9b-mediated mitochondrial elongation and hyperfusion, leading to the appearance of megamitochondria. The ubiquitous expression of ORF6, NSP6, or ORF7a in *Drosophila* muscle cells resulted in a notable decrease in mitochondrial size (Zhu et al., 2021). In addition to affected mitofusion, ORF9b also affects the frequency of mitophagy (fragmented mitochondria, due to DRP1 activity, are directed at lysosomal degradation), however, at the same time, apoptosis can be blocked by S-protein and the decreased level of pro-apoptotic cytokines (Figure 3; Gatti et al., 2020; Malavolta et al., 2020; Madeddu et al., 2022). Yang et al. (2021) suggested that in coronavirus infection, the frequency of autophagy that prevents cells from enhanced apoptosis and maintains cell homeostasis by exchange of damaged mitochondria is markedly reduced in adult patients (but not among children). These responses are associated with a decrease in peripheral blood T cell count among adults with moderate and severe symptoms of COVID-19.

One of the key interactors of ORF9b is the TOM70 complex, which is not only a mitochondrial importin component but also regulates organellar quality and mitochondrial tethering (Figure 3). TOM70 is often present at the ER-mitochondria contact sites. Hampering its activity by ORFf9b leads to direct inhibition of antiviral signaling (Jiang et al., 2020; Kreimendahl and Rassow, 2020). Binding of TOM70 by ORF9b employs hydrophobic interactions between the helical CTD domain (residues 249-608) of TOM70 and the region between 43 and 78 residues of ORF9b. Finally, novel helices are formed that weaken allosteric (mainly electrostatic) interactions between TOM70 and Hsp90 (Gao et al., 2021). For ORF9b-TOM70 interactions, some key residues (S53 in ORF9b, as well as E477 and R192 in TOM70) are particularly important, and phosphorylation regulates interaction of those proteins and affects further Hsp90 recruitment. Substitutions for S53E and R192A weaken those interactions (Brandherm et al., 2021).

## 10 Maintenance of mitochondrial biogenesis in COVID-19

**FIGURE 4:**
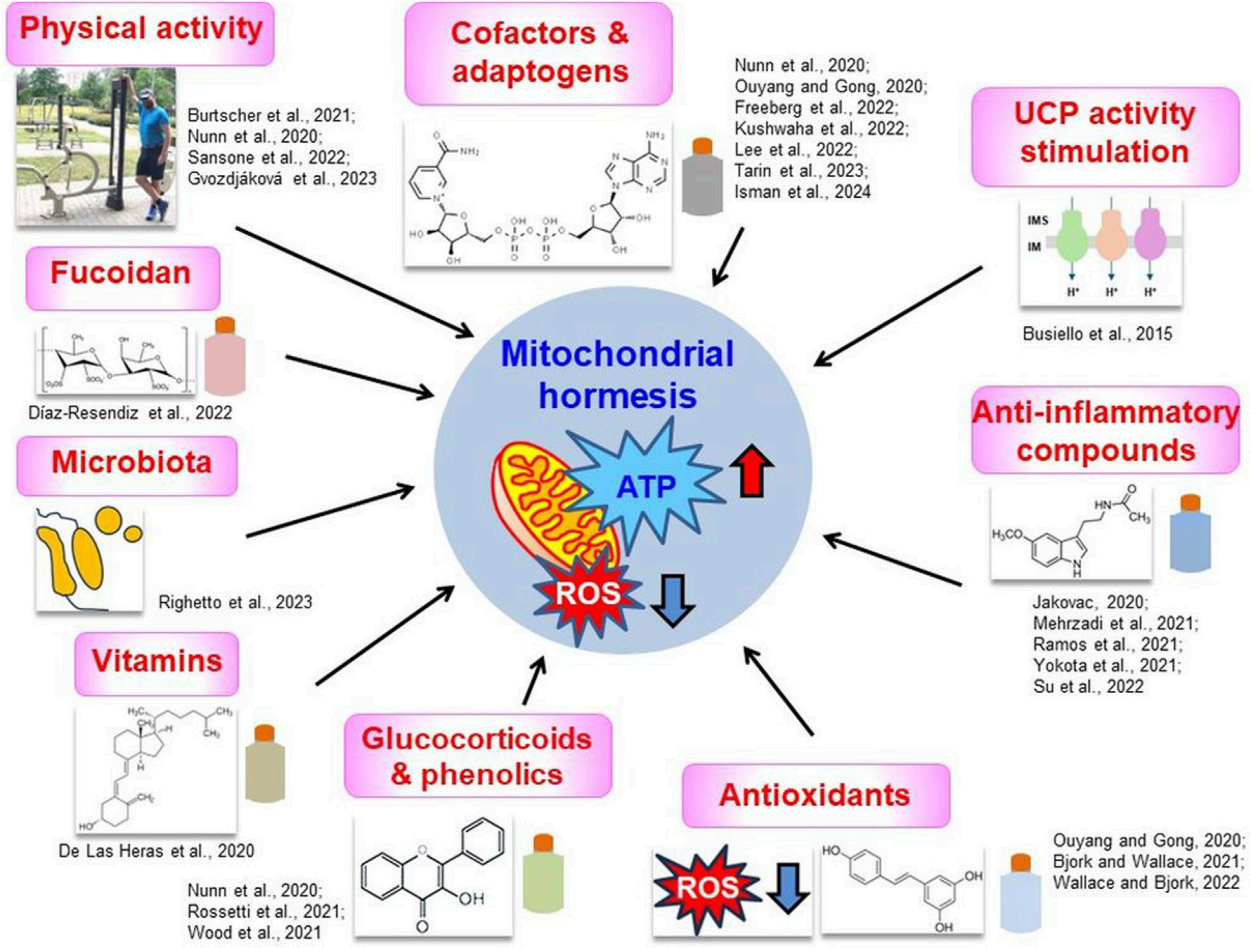
Proposed therapeutic strategies to alleviate COVID-19 symptoms that are often connected with the maintenance of mitochondrial hormesis. Key strategies were shown together with the most important literature data, discussed in the text. Categories of diverse compounds were symbolically depicted as colored flasks. Chemical formulas represent fucoidan (“Fucoidan” panel), vitamin D (“Vitamins” panel), flavonol (“Glucocorticoids and phenolics” panel), resveratrol (“Antioxidants” panel), melatonin (“Anti-inflammatory compounds” panel) and NAD^+^ (“Cofactors and adaptogens” panel). Hormetic mitochondria are characterized by the high efficiency of ATP synthesis and the relatively low ROS level (*red and blue arrows, respectively, in the central panel*). More information in the text. IM, inner membrane; IMS, intermembrane space; ROS, reactive oxygen species; UCP, uncoupling protein. Source of small photo at the “Physical activity” panel: private Author’s collection.

Numerous preventive therapeutic strategies have emerged to alleviate symptoms and protect mitochondrial functionality in COVID-19 (Figure 4). They concern the treatment with a variety of multiple chemical agents, including vitamins, cofactors, and antioxidants that help patients combat COVID-19 and increase the probability of successful recovery and survival.

Among these drugs, remdesivir and molnupiravir interfere with mitochondrial RNA polymerases. At sub-cytotoxic concentrations, no negative impact on mitochondrial biogenesis and bioenergetic parameters was reported (Bjork and Wallace, 2021; Wallace and Bjork, 2022). However, reports on the impact of remdesivir on the quantity of mitogenome are contradictory. Herbst et al. (2022) showed that remdesivir does not alter the number of mitogenome copies or the frequency of deletion in aged male rats; according to more recent reports, however, an increase in the number of mitogenome copies was detected in Mv1Lu and liver cells, although without drastic functional alterations (DeFoor et al., 2023).

NAD^+^ was initially used in various therapeutic applications to alleviate symptoms of cardiovascular, neurodegenerative, and metabolic disorders and to establish its availability as a supplement (Mehmel et al., 2020; Tannous et al., 2021) and was used multiple times to alleviate the consequences of SARS-CoV-2 infection in patients with COVID-19. Lee et al. (2022) proposed using nicotinamide riboside supplementation to reactivate the TCA cycle in COVID-19 patients; it is known that NAD^+^ inhibits viral replication. However, these therapeutic suggestions came from the study that used a viral model that mimicked SARS-CoV-2 infection. Freeberg et al. (2023) used human aortic endothelial cells exposed to plasma from COVID patients and co-incubated them with NAD^+^ precursors, concluding that this treatment hampered COVID-induced reductions in NO production and oxidative stress. Jiang et al. (2022) observed that SARS-CoV-2-induced pneumonia phenotypes were largely rescued after NAD^+^ and its intermediate treatment. Furthermore, Isman et al. (2024) showed that low-dose naltrexone and NAD^+^ supplementation alleviated moderate/severe fatigue among patients with COVID-19.

In combating the severe symptoms of COVID-19, a decrease in aerobic glycolysis rate was suggested, resulting in a high ROS level. Oxidative stress is a very important factor that contributes to the development of chronic fatigue in COVID-19. Therefore, numerous compounds (including vitamin C, phenolics, glucocorticoids, anticancer chemicals, and antioxidants) were proposed to display the alleviating effect under coronavirus infection (Nunn et al., 2020; Rossetti et al., 2021; Wood et al., 2021). Antioxidants, such as resveratrol, decrease the level of superoxide, promote more efficient Ca^2+^ homeostasis, as well as mitophagy, and increase the level of ACE2. Resveratrol has been reported to decrease LDH and HSP60 levels and reduce the frequency of apoptosis in the respiratory system; the latter protein is often translocated from mitochondria and could be secreted from the cell under pro-inflammatory conditions (Jakovac, 2020). Other antioxidant agents, for example, hydroxytyrosol from olive (*Olea europaea*) leaves, may be helpful in modulating the pro-inflammatory response and restricting the pool of ROS; the curcumin and vitamin C, potent antioxidants, provide general antiviral and anti-inflammatory protection, increase IFN production and inhibit cytokine release, respectively. Additionally, mitochondrially targeted ubiquinone, as antioxidant, improves mitochondrial dysfunctions in various diseases; Ouyang and Gong (2020) proposed to use this compound in a potential treatment or within adjunctive therapy of COVID-19. They speculated that it could improve T cell performance and decrease the “cytokine storm.” Furthermore, Kushwaha et al. (2022) developed a nanocurcumin-based formulation using pyrroloquinoline quinone that protects cardiopulmonary function and mitochondrial homeostasis under hypoxia in animal models and human ventricular cardiomyocytes; this compound was also proposed to be promising in COVID-19 therapy.

Another systemic antioxidant and anti-inflammatory agent, melatonin, stimulates the activity of the TOM/TIM complex, affects mitochondrial fission (owing to antioxidative properties and the prevention of cellular Ca excess), and thus positively influences mitochondrial quality control leading to the improvement of mitochondrial functions (Mehrzadi et al., 2021; Yokota et al., 2021). Its importance can be highlighted by the fact that the level of melatonin decreases with age and is related to the current physiological state of mitochondria. Thus, decreased melatonin levels are associated with the risk of a severe onset of COVID-19 as well as mitochondrial dysfunctions. In general, melatonin supplementation in elderly people was shown to be beneficial in maintaining mitochondrial hormesis and its alleviation role in preclinical and clinical studies of respiratory diseases was confirmed (Ramos et al., 2021; Su et al., 2022).

Vitamin D could “seal” biological membranes and increase the immune response during coronavirus infection. According to De Las Heras et al. (2020), vitamin D deficiency appears to be associated with an increased risk of COVID-19. This compound can normalize mitochondrial dynamics, which would alleviate oxidative stress and inflammation. Furthermore, vitamin D reduces activation of the renin-angiotensin-aldosterone system and decreases the ROS level. Other compounds, such as flavanols, including quercetin, induce autophagy (and thus prevent mitochondrial hyperfusion); in general, they can also inhibit interactions between SARS-CoV-2 and the ACE2 receptor. Thus, quercetin galactoside becomes a promising ACE2 inhibitor. As Zn^2+^ regulates the “cytokine storm,” it was suggested that it can inhibit SARS-CoV-2 replication by decreasing RNA binding by polymerase and inhibiting the replication elongation step. Amylorid and methylene blue also inhibit SARS-CoV-2 replication; furthermore, amylorid regulates cellular K^+^ transport and delays the “cytokine storm” (Nunn et al., 2020). Variations in Cu^2+^ level may also be pronounced under COVID-19, and recently elesclomol, a copper-binding agent that targets mitochondria, was proposed as a therapeutic drug in COVID-19 (Tarin et al., 2023).

The stimulation of uncoupling proteins (e.g., UCP3) leading to the general stimulation of metabolism in obesity and diabetes (Busiello et al., 2015) may also be prospective in combating the consequences of SARS-CoV-2 infection. Furthermore, agonists of the sigma-1 receptor may protect mitochondria and inhibit ER stress, manage Ca^2+^ homeostasis, and, in general, induce autophagy to prevent cell death in response to infection (Brimson et al., 2021).

Other studies indicated that some polysaccharides (for example, fucoidan) could decrease the potential of the mitochondrial membrane in peripheral blood monocytes among COVID-19-rescued patients. Díaz-Resendiz et al. (2022) suggested that fucoidan therapy mitigates effects of “long COVID-19.” Surprisingly, Aguida et al. (2023) showed that near-infrared light stimulates mitochondrial metabolism, inducing a noticeably short ROS burst, as well as an anti-inflammatory response, indicating a potential novel therapeutic method for SARS-CoV-2 infections.

The gut microbiota can be a source of signals, transmitted through sensory neurons innervating the gut, able to influence brain structure and function, including cognitive functions. There is also a link between the microbiota and mitochondrial status and susceptibility to COVID-19 infections (Righetto et al., 2023). Intestinal microenvironmental dysfunctions can modulate the occurrence of COVID-19 by also affecting mitochondrial functions in the intestinal epithelium, and to avoid this transplantation of fecal bacteria, prebiotic and probiotic supplementation is warmly recommended (Wang et al., 2021).

Finally, one of the most important factors that prevents the severe outcome of COVID-19 is the hormetic lifestyle, with numerous physical activities that allow the activation of OXPHOS, which does not favor the propagation of SARS-CoV-2 (Burtscher et al., 2021). Gvozdjáková et al. (2023) noted that mountain spa rehabilitation improved platelet mitochondrial respiration among patients after COVID-19. To keep mitochondria hermetic, it would be also important to avoid intake of any harmful drugs. For example, nicotine intake together with SARS-CoV-2 infection affect mitochondrial biogenesis, resulting in swelling of mitochondria and other ultrastructural aberrations, increasing various IL and TNF levels, as well as leading to extended pyroptosis and necroptosis (Sansone et al., 2022).

Recently, multiple strategies based on genetic modification of human cells or mitochondrial targeting were alternatively proposed; two of them will be briefly described here. A very prospective approach concerns the transfer of mitochondria overexpressing MAVS components, by genetic modification of mesenchymal stem cells to treat severe cases of COVID-19 (Babajani et al., 2021). Modified stem cells enter the walls of blood vessels into the alveolar epithelium, modulate the immune response controlling the cytokine storm, affect macrophage biogenesis, help clean the inside of secretions alveoli, promote tissue regeneration (protection against fibrosis), reduce the level of angiotensin II, and reduce the risk of endovascular clots. Mesenchymal stem cells that express S-protein and MAVS-overproducing, transfer the transgenic mitochondria to alveolar cells. The activated RIG-I and MDA-5 proteins could then bind to the MAVS target on the OMM of transferred donor mitochondria. This further activates NF-kB and IRF factors that can induce the expression of genes related to the immunological response against SARS-CoV-2 and the IFN synthesis could start. This strategy allows the MAVS signalosome to be activated, even though the remaining unmodified mitochondria are still hijacked by the coronavirus (Babajani et al., 2021). Recently, Logue et al. (2023) targeted 2′,3′cyclic nucleotide 3′-phosphodiesterase (CNP), an inhibitor of β-coronavirus replication, in mitochondria and showed that it is necessary to inhibit SARS-CoV-2 biogenesis, implicating the proposed role of CNP as an inhibitor of mitochondrial permeabilization transition pore as the mechanism of inhibition of virion assembly.

## 11 Conclusion and future directions

Under SARS-CoV-2 infection, drastic reprograming of mitochondrial biogenesis by hijacking organellar signaling is very deep. Most of those responses are summarized in Figure 3, which depicts a particularly complex pattern of interactions between the genome fragments and proteins of SARS-CoV-2 and the host mitochondrial proteins, resulting in the halting of numerous mitochondrial activities. Multiple structural and non-structural proteins of SARS-CoV-2 influence those processes, both directly and indirectly. As COVID-19 belongs to polysystemic diseases, mitochondrial biogenesis and respiratory metabolism can be affected in various cells, e.g., respiratory tracts, brain neurons and glial cells, kidneys, liver, blood cells and serum, placenta, epithelium, *etc.* Numerous cell types present in these tissues require highly active mitochondria for their functionality. Therefore, it should be noted that focusing only on respiratory responses cannot guarantee a broad perspective on the consequences of SARS-CoV-2 infection in the mitochondrial compartment.

During SARS-CoV-2 infection, the functions of MAVS as the central signalosome complex are altered, primarily by inhibiting RLR. MAVS regulates antiviral signaling related to NF-kB activation, IFN induction, and entry into the autophagy pathway. Β-coronaviruses, including SARS-CoV-2, inhibit IFN production by interfering with various signaling pathways, including MAVS and STING proteins. In general, deep alterations occur in the innate immune response, leading to a chronic inflammatory state, which is further deepened by multiple mitochondrial aberrations. In addition, mitochondrial senescence contributes to the severe cases of COVID-19 and to the continuous activation of the inflammasome and permanent oxidative stress. During SARS-CoV-2 infection, OXPHOS activity is hampered and, inversely, the anaerobic glycolytic rate increases (as oxygen level is reduced under severe hypoxemia), and efficient ATP production is affected. Additionally, cellular calcium and iron homeostasis is severely affected and leads to downstream consequences; for example, the increased level of free iron within mitochondria is due to pro-inflammatory processes, and iron overaccumulation promotes further oxidative stress, leading to lipid peroxidation and a decrease in glucose tolerance level. SARS-CoV-2 also affects mitochondrial dynamics, motility, and membrane system quality control by fission and mitophagy repression and favors mitochondrial hyperfusion. Therefore, mitochondrial homeostasis is severely affected by SARS-CoV-2 infection. Numerous subgenomic SARS-CoV-2 RNA fragments are assumed to be present in the membrane continuum of host cells and mitochondria have been speculated to be involved in virus replication (Shang et al., 2022). In COVID-19, the mitogenome mutation rate increases and mitogenome fragments can be sensed through multiple signaling cascades and together with some mitochondrial proteins they can be used as prospective biomarkers for the assessment of COVID-19 severity.

The actual physiological status of mitochondria influences the response of these organelles to SARS-CoV-2 infection. To maintain mitochondrial hormesis during acute SARS-CoV-2 infections, multiple preventive strategies were proposed, including the supplementation of low molecular compounds, vitamins, phenolics, polysaccharides, cofactors, anticancer, antioxidative and anti-inflammatory agents to increase the relevance of oxidative phosphorylation during the mitigation of symptoms of COVID-19. Emerging evidence on the role of the gut microbiota status in the pathogenesis of COVID-19 is also collected. Finally, one of the most important factors in preventing the severe outcome of COVID-19 is a good lifestyle, with extensive physical activities.

Based on the existing research on COVID-mitochondrial relationships and interdependencies, few areas need to be studied in more detail: (1) links between the level of selected non-coding RNA and alterations in the expression pattern of genes regulating mitochondrial biogenesis; (2) mitochondrial interactome network involved in COVID-19 pathogenesis; (3) mitochondrial biomarkers associated with COVID-19 severity; (4) assays for screening of patients with COVID-19, based on the detection of mitochondrial protein activity or abundance, and (5) mitochondria targeting methods employing nucleic acids, such as, for instance, RNA-based therapeutics (Yamada et al., 2022).

## Figures and Tables

**Table:** 

**ACADM**	acyl-CoA dehydrogenase medium chain
**ACE**	angiotensin-converting enzyme
**AhR**	aryl hydrocarbon receptor
**AID4E3**	HECT domain E3 ligase
**AIM**	absent in melanoma
**Ang**	angiotensin
**ANT**	adenine nucleotide translocator
**ASC**	apoptosis-associated speck-like protein containing a CARD
**ATP1B1**	ATPase Na^+^/K^+^ transporting subunit β1
**ATP6V1A**	ATPase H^+^ transporting V1 subunit A
**Bcl-XL**	B cell lymphoma-extra-large
**BOK**	B cell lymphoma ovarian killer
**CI**	complex I
**CIII**	complex III
**CCDC**	coiled-coil domain containing
**cGAS**	cyclic GMP–AMP synthase
**CM**	coiled membrane
**CNP**	2′,3′cyclic nucleotide 3′-phosphodiesterase
**CO/COX**	cytochrome *c* oxidase (complex IV) subunit
**CoQ**	coenzyme Q
**COQ8B**	coenzyme Q8B
**COVID**	coronavirus disease
**CT**	cytoplasmic tail
**CTD**	C-terminal domain
**CTLH**	C-terminal to LisH
**CYT**	cytochrome *c* oxidase (complex III) subunit
**CYTB**	cytochrome *b*
**DMV**	double membrane vesicle
**DNM1L**	dynamin-1-like protein
**Drp**	dynamin-related protein
**EB**	Epstein-Barr
**E-protein**	envelope protein
**ERLIN**	endoplasmic reticulum lipid raft associated
**Fis**	fission
**FP**	fusion peptide
**G3BP1**	Ras GTPase-activating protein-binding protein 1
**GIGYF**	GRB10-interacting glycine-tyrosine-phenylalanine domain
**HECT**	homologous to the E6-AP carboxyl terminus
**HEK**	human embryonic kidney
**HF**	hemagglutinin esterase
**HIF**	hypoxia inducible factor
**HMOX**	heme oxygenase
**hnRNPs**	heterogenous ribonucleoprotein particles
**HPMEC**	human pulmonary microvascular endothelial cells
**HR**	heptad repeat
**Hsp**	heat shock protein
**HUVAC**	human umbilical vein endothelial cells
**IDO**	indoleamine 2,3-dioxygenase
**IFN/Ifn**	interferon
**IL**	interleukin
**IMM**	inner mitochondrial membrane
**IP3R**	inositol trisphosphate receptor
**ISG**	interferon-stimulated gene
**JAK-STAT**	Janus kinases- signal transducer and activator of transcription proteins
**LDH**	lactate dehydrogenase
**LFA**	lymphocyte function-associated antigen
**L2HGDH**	L-2-hydroxyglutarate dehydrogenase
**lncRNA**	long non-coding RNA
**MAM**	mitochondria-associated membrane
**MAVS**	mitochondrial anti-viral signaling
**MCL**	myeloid cell leukemia
**MDA**	melanoma differentiation-associated protein
**MERS**	Middle East respiratory syndrome
**Mff**	mitochondrial fission factor
**Mfn**	mitofusin
**mitomiR**	microRNA regulating mitochondrial biogenesis
**mitomiR**	miRNA for mitochondrial biogenesis
**MOTS-c**	short mitochondrial peptide encoded in the mitogenome
**M-protein**	membrane protein
**Mrg**	Mas-related genes
**MRP**	multidrug resistance protein
**MRPL**	mitochondrial ribosome large subunit protein
**mTOR**	mechanistic target of rapamycin
**mtPTP**	mitochondrial permeability pore
**Mv1Lu**	cell line
**Myd**	myeloid differentiation primary response
**ncRNA**	non-coding RNA
**ND**	NADH-dehydrogenase (complex I) subunit
**NDUF**	complex I subunit
**NDUFAF**	complex I assembly factor
**NF-κB**	nuclear factor kappa-light-chain-enhancer of activated B cells
**NIM**	N-methyl-4-isoleucine cyclosporin (mitochondrial permeability transition inhibitor)
**NIX**	Nip3-like protein X
**NK**	natural killer
**NLRP**	nucleotide-binding oligomerization domain, leucine rich repeat and pyrin domain containing
**NLRX1**	nucleotide-binding oligomerization domain, leucine rich repeat containing X1
**NOD**	nucleotide-binding oligomerization domain
**N-protein**	nucleocapsid protein
**NSP**	non-structural protein
**NTD**	N-terminal domain
**OMM**	outer mitochondrial membrane
**OPA**	mitochondrial dynamin like GTPase
**OXPHOS**	oxidative phosphorylation
**PASC**	post-acute sequelae of COVID-19
**PCBP**	poly(rC)-binding protein
**PCD**	programmed cell death
**PHB**	prohibitin
**PINK**	PTEN-induced kinase
**PITRM**	pitrilysin metallopeptidase
**PKM**	pyruvate kinase muscle
**PLpro**	papain-like protease
**PMP**	peptidase mitochondrial processing subunit
**Pp**	polyprotein
**PRF**	programmed ribosomal frameshift
**PTP**	permeability pore
**RBD**	receptor-binding domain
**RIG**	retinoic acid-inducible gene
**RLR**	RIG-I-like receptor
**RNF**	ring-finger protein
**SARS-CoV**	severe acute respiratory syndrome-coronavirus
**SDH**	succinate dehydrogenase
**sgRNA**	subgenomic RNA fragments
**SOD**	superoxide dismutase
**SPG**	spastic paraplegia, paraplegin
**S-protein**	‘spike’ protein
**STING**	stimulator of interferon genes
**STOML**	stomatin-like
**TCA**	tricarboxylic acid cycle
**TFAM**	mitochondrial transcription factor A
**TIM**	translocase of the inner membrane
**TLR**	Toll-like receptor
**TM**	transmembrane domain
**TMPRSS**	transmembrane serine protease
**TNF**	tumor necrosis factor
**TO(M)M**	translocase of the outer (mitochondrial) membrane
**UCP**	uncoupling protein
**UTR**	untranslated region
**VDAC**	voltage-dependent anion channel
